# Measuring resilience after spinal cord injury: Development, validation and psychometric characteristics of the SCI-QOL Resilience item bank and short form

**DOI:** 10.1179/2045772315Y.0000000016

**Published:** 2015-05

**Authors:** David Victorson, David S. Tulsky, Pamela A. Kisala, Claire Z. Kalpakjian, Brian Weiland, Seung W. Choi

**Affiliations:** 1 Department of Medical Social Sciences, Northwestern University Feinberg School of Medicine, Chicago, IL, USA; 2 Department of Physical Therapy, College of Health Sciences, University of Delaware, Newark, DE, USA; 3 Kessler Foundation Research Center, West Orange, NJ, USA; 4 Department of Physical Medicine and Rehabilitation, University of Michigan, Ann Arbor, MI, USA; 5 CTB/McGraw Hill, Monterey, CA, USA

**Keywords:** Patient outcomes assessment, Psychological resilience, Psychometrics, Quality of life, Spinal cord injuries

## Abstract

**Objective:**

To describe the development and psychometric properties of the Spinal Cord Injury - Quality of Life (SCI-QOL) Resilience item bank and short form.

**Design:**

Using a mixed-methods design, we developed and tested a resilience item bank through the use of focus groups with individuals with SCI and clinicians with expertise in SCI, cognitive interviews, and item-response theory based analytic approaches, including tests of model fit and differential item functioning (DIF).

**Setting:**

We tested a 32-item pool at several medical institutions across the United States, including the University of Michigan, Kessler Foundation, the Rehabilitation Institute of Chicago, the University of Washington, Craig Hospital and the James J. Peters/Bronx Department of Veterans Affairs medical center.

**Participants:**

A total of 717 individuals with SCI completed the Resilience items.

**Results:**

A unidimensional model was observed (CFI = 0.968; RMSEA = 0.074) and measurement precision was good (theta range between −3.1 and 0.9). Ten items were flagged for DIF, however, after examination of effect sizes we found this to be negligible with little practical impact on score estimates. The final calibrated item bank resulted in 21 retained items.

**Conclusion:**

This study indicates that the SCI-QOL Resilience item bank represents a psychometrically robust measurement tool. Short form items are also suggested and computer adaptive tests are available.

## Introduction

Traumatic spinal cord injury (SCI) is an unexpected, life-altering event that changes a person's life in an instant, often as the result of a motor-vehicle accident, violence, or fall.^1^ Individuals with traumatic SCI are exposed to a highly distressing and potentially debilitating set of circumstances related to limitations in motor and sensory functioning and psychological trauma.^2^ Traumatic injuries often occur when individuals are young and in their prime, significantly disrupting the normal, developmental trajectory of their lives.^3^ As a result of injury and subsequent disability, many activities and employment opportunities that had been a source of pleasure and life satisfaction become restricted or no longer possible,^4^ while new types of recreational, leisure, and vocational activities must be learned.^3^ After injury, there is an increased incidence of depressive disorders,^5–9^ anxiety disorders,^10^ post-traumatic stress disorder,^11^ and other forms of psychological distress and adjustment problems. Moreover, many individuals with SCI often rate their life satisfaction^12–14^ and quality of life^15^ significantly lower compared to those without disabilities. Despite this, some individuals with SCI are also able to successfully adapt to these stressors and maintain a sense of psychological well-being and stability in the face of such adversity.^16–18^ In fact, individuals who can ‘bounce back’ from highly stressful life events seem better able to flexibly co-experience both negative affect-based emotional states alongside positive affect and eudaimonic states (e.g. deriving meaning and purpose beyond basic self-gratification).^19^

Because of the dramatic and sudden onset of traumatic SCI, investigators and clinicians have long been interested in adaptation to life after injury.^20,21^ The concept of resilience is particularly important in this population given the wide-ranging impact of injury on physical, psychological, and social functioning. In the psychological literature, resilience has been defined and characterized as many things – as a fixed trait,^22^ as a developable state,^23^ as an ability,^24^ as a defense mechanism,^25^ as a dynamic process,^26^ and as an outcome, all of which are similarly characterized by adaptive and flexible responses in the face of highly stressful life events.^27^ In their review of resilience measurement scales, Windle *et al*.,^28^ acknowledged the difficulty of defining this complex construct and proposed a multilevel definition of resilience as the process of successfully adapting to significant sources of stress or trauma, facilitated by an individual's psychological resources, life experiences, and environment. Notwithstanding, the concept of resilience lacks a common theoretical framework, which has resulted in inconsistencies in measurement and the identification of risk and protective factors across different studies.^25,28^

In the context of SCI, resilience is negatively associated with depression and anxiety, and positively associated with subjective well-being.^29–31^ In work to understand the process of adjustment after injury, four trajectories are described by Bonnano and Quale: (1) *resilient*, in which individuals maintain or quickly return to a healthy psychological state soon after the event; (2) *recovery*, in which symptoms of distress may reach threshold or sub-threshold levels of psychopathology (e.g. depression, anxiety) but gradually subside over a period of months or years; (3) *delayed distress*, in which symptoms of distress gradually worsen over time; and (4) *chronic dysfunction*, in which individuals struggle for many years.^16,32^ Bonanno *et al.*^16^ specifically found that a majority of individuals reported a continuous, stable, low symptomatic response characteristic of a resilient trajectory. Around 25% exhibited the recovery trajectory and only 12.5 and 12.8% displayed the chronic high depression and delayed depression trajectories, respectively. Acknowledging the limitation of approaching resilience as the absence of psychopathology, this study also showed that the resilient trajectory group showed fewer SCI-related quality of life problems than either the chronic or delayed depression groups.

Social support is associated with higher reported levels of life satisfaction following SCI, particularly in individuals with less functional independence.^33^ Social cognition, sense of coherence, locus of control, and sense of purpose in life have all been identified as additional factors that may have a role in adaptive coping in individuals with SCI.^34,35^ In contrast, negative affect, comorbid psychiatric illness, and feelings of isolation have been identified as risk factors for poorer long term adjustment.^32^

There are numerous resilience measurement instruments for children, adolescents and adults, but there is no established gold standard.^28^ Measures include the Adolescent Resilience Scale,^36^ the Resilience Scale of the Student Survey,^37^ the Dispositional Resilience Scale,^38,39^ scales of Ego Resiliency,^40–42^ the Connor-Davidson Resilience Scale (CD-RISC),^43,44^ the Youth Resiliency Scale,^45,46^ the Resilience Scale for Adults,^47,48^ the Resiliency Attitudes and Skills Profile,^49^ the Brief Resilience Scale,^50^ the Child and Youth Resilience Measure,^51^ the Resilience Scale,^52^ the scale of Psychological Resilience,^53^ and the Resilience Scale for Adolescents.^54^ Common across scales are dimensions of hardiness, such as commitment, control and challenge; perceived competence across personal and social structures; emotional regulation and interpersonal control; optimism; and positive acceptance. Compounding the absence of a gold standard, different operational definitions across these instruments highlight the confusion resulting from the absence of a unifying construct and the difficulty of comparing studies utilizing these instruments.

Moreover, none of these instruments was developed or tested specifically for use in individuals with SCI, although some recent work has used standardized instruments like the CD-RISC in SCI samples.^29,30^ Nevertheless, much of the work in resilience has been in children and adolescents and focused on developmental transitions of greatest stress.^24,55^ In contrast, SCI is neither expected nor a developmental transition. Instead, it is a highly disruptive event that pervasively impacts physical, psychological, and social functioning. Moreover, cross-cultural studies of resilience demonstrate that it is not a universal, homogenous construct, but rather that certain processes or traits may be more crucial to adapting to trauma depending on the individual's physical and social contexts.^56^ Similarly, work to understand adjustment to injury has also shown that an individual's resources – psychological, social, and functional – play an important role in adaptation after injury.^57–59^

As detailed by Tulsky *et al.* in the introductory paper of this issue,^60^ recent federally funded efforts have worked to improve and standardize the measurement of patient reported outcomes in the general population^61^ and within specific disease groups.^62^ To our knowledge, no efforts to date have attempted to utilize similar modern measurement approaches to enhance the measurement of patient reported outcomes following SCI. More specifically, no scales currently exist that measure resilience in the SCI population. Understanding adjustment processes and the role of resilience after SCI has important implications for clinical approaches to facilitate adaptation and quality of life. Thus, the purpose of this paper is to present findings from the development and psychometric calibration of the SCI-QOL Resilience item bank and short forms.

## Methods

This study was approved by all participating sites' Institutional Review Boards. A first study activity was to develop and refine a resilience item pool. Next, resilience items were administered to a large sample of people with SCI using a computerized data collection platform and interview format, so that each question was read to the respondent by a trained interviewer and responses were directly entered into the database. Each of these steps is described in detail in Tulsky *et al.* (this issue)^60^ and is also outlined briefly in the section below.

### Development of a resilience item pool

To guide the development of a new resilience measurement tool in SCI, we adopted the operational definition of resilience by Windle *et al.*^28^, as the process of successfully adapting to significant sources of stress or trauma, facilitated by an individual's psychological resources, life experiences, and environment. Next, we began by identifying candidate items from our initial pilot work, which included individual, semi-structured interviews with individuals with SCI. Interview participants were placed in the role of ‘expert’ and asked to generate topics and items that should be included in a measure of HRQOL for individuals with SCI. Next, individuals with SCI and SCI clinicians separately participated in a series of focus groups which examined the impact of SCI on HRQOL (see Tulsky *et al.*^63^ for a full description). From these data we developed a set of 1118 preliminary items related to emotional health and functioning, of which 61 were ultimately binned into the ‘Resilience’ subdomain. For example, from the patient focus group quote, ‘*I've always been an advocate no matter what a person's situation is. There's always a positive side of the situation*’ we drafted the item, ‘I try to see the positive side of things.’ After item review and modification for grammatical tense and consistency, this was subsequently changed to, ‘I tried to see the positive side of things.’ An individual interview statement, ‘*I have accepted my SCI and how it has impacted my life*’ was used to draft the item, ‘I accept my SCI,’ which was later modified to ‘I have accepted my injury.’ The initial 61 items underwent Expert Item Review (EIR),^64^ a method whereby several project co-investigators reviewed each item for relevance and clarity and made suggestions for revisions and deletions. Based on EIR feedback, 22 items were deleted, 3 were moved to a different domain (social participation), and 36 were retained in the preliminary Resilience item pool. These items then underwent an additional phase of item review and modification by members of the investigative team. Items were arranged on a continuum from items indicating the lowest amount of resilience to the highest amount of resilience. Team members removed redundant items where there was oversaturation in the middle range of the hierarchy, and suggested new items to fill gaps in content coverage. Specifically, 18 items were deleted during this phase of review, and 15 new items were added. One of the study team members (author DV) had recently developed the Injury Resilience Index (IRI),^65^ a measure of resilience specifically for individuals who had sustained traumatic physical injuries. All 19 IRI items were reviewed for content, and 9 modified items (e.g. ‘*How I am affected by the accident is an opportunity for growth*’ became ‘*I felt my injury is an opportunity for personal growth*’) were selected for inclusion in the SCI-QOL. An additional 5 items were re-binned into the Resilience pool following review of other subdomains (e.g. Positive Affect & Well Being, Grief/Loss), and one entirely new item was drafted by the study team.

This refined set of 33 items was then pre-tested with individuals with SCI during structured cognitive debriefing interviews,^66^ in which respondents were asked to answer each item, then describe the process they used to come up with their answer and relate whether they perceived anything to be confusing, unclear, or derogatory, or whether they thought any items could be better phrased. Five items were modified and one was deleted based on cognitive interviewing. After this phase, the final 32 items were reviewed for translatability (for method, please see Eremenco *et al.*^67^) and reading level (using the Lexile framework^68^). Slight modifications were made to three items after the translatability and cultural review. For example, the item ‘my *spirituality* strengthened me’ was changed to ‘my *faith* strengthened me,’ since translation to the word ‘*espiritualidad*’ would not make sense in this context, and the item ‘I am able to bounce back from challenging situations pretty well’ was changed to ‘I was able to handle difficult situations' given the colloquialism of the term ‘bounce back’. All items were written at the 5th grade reading level.

### Calibration study participants and data collection procedures

As a part of a large-scale multisite item calibration study (sites included the Kessler Foundation, University of Michigan, Rehabilitation Institute of Chicago, University of Washington, Craig Hospital and the James J. Peters/Bronx Department of Veterans Affairs hospital), we administered the 32 resilience items along with other item pools reflecting different HRQOL subdomains to a sample of people with SCI. The calibration sample included 717 participants with SCI. Inclusion criteria were 18 years of age and older, ability to read and understand English, and medically documented traumatic SCI. The sample was stratified by level (paraplegia versus tetraplegia), completeness of injury (complete vs. incomplete), and time since injury (<1 year, 1–3 years, and >3 years) to ensure that the final sample was a heterogeneous sample of individuals with SCI. Each participant's diagnosis was confirmed by medical records and each participant's neurologic level was documented by their most recent American Spinal Injury Association Impairment Scale (AIS) rating.^69^ All items were presented in a structured interview to participants in person or over the phone. The methodology for this study is presented in detail in Tulsky *et al.* (this issue)^60^ and will not be repeated here.

### Data analyses

Analysis involved confirmation of construct unideminsionality, use of a graded-response item response theory (IRT) model to calibrate item parameters, and examination of differential item functioning (DIF). We used confirmatory factor analyses to determine if our items conformed to a unidimensional model. Criteria for acceptable model fit were as follows: CFI > 0.90, RMSEA < 0.08, good; CFI > 0.95, RMSEA < 0.06, excellent. Calibration was performed using iterative methods to refine the item pool and obtain the best-fitting item parameters that would allow accurate estimation of a participant's standing on a trait of resilience. With each successive analytic iteration, we identified poorly fitting items by examining item fit to the graded-response IRT model, DIF, local dependence between items (residual correlations >|0.20|), and non-significant loadings on the single factor (values <0.30). We then removed these items from the item pool and repeated the analytic steps. Once an acceptable solution was reached with CFA statistics that supported a unidimensional model, and all items showing misfit to the IRT model or DIF were removed, the final IRT parameters were utilized to develop CAT algorithms for the Resilience item bank. The CAT was programmed on the Assessment Center website (http://www.assessmentcenter.net) and can be administered directly from the website. The final IRT parameters were also used to select items for a static short form which can also be downloaded as a PDF from the Assessment Center website.

### Reliability study

The SCI-QOL measures were administered to a separate sample of individuals with traumatic SCI as part of a larger study.^70^ Participants completed the SCI-QOL Resilience CAT at baseline and 1–2 weeks. Pearson's *r* and the intraclass correlation coefficient (ICC) were to assess test-retest reliability.

## Results

### Participant characteristics

Resilience, depression, anxiety, self-esteem items and other items were administered to a calibration sample of 717 individuals with SCI. Demographic and injury characteristics are summarized in Table 1. Please see Tulsky *et al*. methodology paper^70^ within this special issue for additional socio-demographic and clinical details on the calibration sample.

**Table 1:** Calibration sample demographics

Variable	Emotional domain sample, *N* = 717; Mean (SD), *N* (%)
Age (years)	43.0 (15.3)
Age at injury (years)	36.1 (16.8)
Sex	
Male	559 (78%)
Female	158 (22%)
Ethnicity	
Hispanic	82 (11%)
Non-Hispanic	631 (88%)
Not reported/Refused	4 (1%)
Race	
Caucasian	505 (70%)
African-American	125 (17%)
Asian	8 (1%)
American Indian/Alaska Native or Native Hawaiian/Pacific Islander	7 (1%)
More than one race	9 (1%)
Other	50 (7%)
Not provided/Refused	4 (1%)
Time since injury	7.1 (10.0)
<1 year post injury	196 (27%)
1–3 years post injury	186 (26%)
>3 years post injury	335 (47%)
Diagnosis	
Paraplegia complete	182 (25%)
Paraplegia incomplete	143 (20%)
Tetraplegia complete	157 (22%)
Tetraplegia incomplete	231 (33%)
Unknown	4 (0%)

The reliability sample consisted of 245 community-dwelling individuals with SCI who had been injured greater than one year at the time of baseline assessment. Additional detail on this sample may also be found in Tulsky *et al.* (this issue).^70^

### Preliminary analysis and item removal

Following initial CFA/IRT analysis we removed 11 items from the tested 32-item pool, first reducing it to 26 items, and finally to 21 items. Four of these items misfit the model (e.g. significant *χ*^2^), had low *R*^2^, and demonstrated local dependence with other items. These four items focused on aspects of perceived growth (e.g. *I felt my injury was an opportunity for personal growth; I felt my injury will help me to change in positive ways*) or use of faith/spirituality (e.g. *my faith strengthened me*). A second group of items was removed because of low *R*^2^ and local dependence. Examples of these items focused on goal attainment (e.g. *I was able to meet personal goals; I believed my injury would make it difficult for me to achieve my goals*) and active coping (e.g. *I did things that would help my rehabilitation*).

After removal of these 11 items, we examined internal consistency (Cronbach's alpha), corrected item-total correlations, the existence of excessive missing data (missing responses for greater than 5 items), sparse cells (due to sample size, fewer than 5 responses), and violations of monotonicity. For the 21 items, alpha coefficient was high (*α* = 0.95) and item/total correlations ranged from 0.54 to 0.78. All of the items had more than 20% of the sample selecting category 5 (Always or Never for reversed item) and no cases were deleted due to excessive missing data. Additionally, no items had sparse data (fewer than 5 responses) and only one item (*I had given up on myself*) had a category inversion with the average raw score for persons selecting category 2 (Rarely) were lower than the average for person selecting category 1 (Never). No additional items were removed at this stage. The following results are based on the final 21 item set.

### Dimensionality

Using CFA, a unidimensional model was observed (CFI = 0.968; RMSEA = 0.074). *R*^2^ values for 20 of the items were greater than 0.40, with one *R*^2^ value slightly less than 0.40 (*I found new ways to solve problems*: *R*^2^ = 0.389). No item pairs evidenced local dependence (i.e. residual correlations >|0.20|).

### IRT parameter estimation and model fit

Slopes ranged from 1.48 to 3.26 while thresholds ranged from –3.26 to 1.11 (see Table 2).

**Table 2:** Resilience items and item bank parameters

		Item response theory calibration statistics				
Item ID	Item stem	Slope	Threshold 1	Threshold 2	Threshold 3	Threshold 4
Resilience_32	I had given up on myself	1.75272	−3.19449	−2.28929	−1.27729	−0.49044
Resilience_6	I was able to manage my life	1.75342	−2.74088	−1.88373	−0.66215	0.46579
Resilience_2	I felt motivated	2.29586	−2.52889	−1.75347	−0.50769	0.56102
Resilience_27	I strived to improve myself	1.79322	−3.26074	−2.43580	−1.19054	−0.03114
Resilience_26	I was able to recognize and overcome challenges	2.70552	−2.74469	−1.83458	−0.57236	0.63103
**Resilience_9**	**I tried to see the positive side of things.**	**2.78396**	**−2.77487**	**−2.07635**	**−0.96070**	**0.10671**
Resilience_31	I found new ways to solve problems	1.47516	−2.88794	−1.95879	−0.32703	1.10999
Resilience_12	I could adapt easily to new situations	1.97179	−2.92753	−1.84342	−0.42858	0.82820
**Resilience_25**	**I was confident that I could overcome my limitations.**	**2.49033**	**−2.29061**	**−1.49300**	**−0.46195**	**0.60415**
Resilience_24	I was able to handle difficult situations	2.38919	−2.71758	−1.85479	−0.53996	0.64778
Resilience_29	I was happy with my ability to cope with my injury	2.92095	−2.22885	−1.51770	−0.52341	0.48801
**Resilience_11**	**I found new things to enjoy.**	**1.89894**	**−2.33796**	**−1.21427**	**0.10162**	**1.01527**
**Resilience_23**	**I felt I can get through difficult times.**	**2.86073**	**−2.81539**	**−1.87238**	**−0.63210**	**0.40837**
**Resilience_10**	**I had a positive attitude.**	**3.26283**	**−2.67671**	**−1.66914**	**−0.70469**	**0.33249**
Resilience_14	I was driven to succeed in my life	2.38527	−2.23904	−1.47524	−0.45307	0.43582
**Resilience_5**	**I used positive ways to cope with my injury.**	**3.05822**	**−2.63899**	**−1.79753**	**−0.62957**	**0.37740**
Resilience_20	I felt the things I went through made me a stronger person	1.89313	−2.57051	−1.70510	−0.77657	0.12463
Resilience_3	I accepted my injury	1.56222	−2.11446	−1.66458	−0.85222	0.10169
Resilience_30	I achieved emotional balance in my life	2.21132	−2.41595	−1.47718	−0.36837	0.82804
**Resilience_7**	**I felt good about how I have coped with my injury.**	**3.04441**	**−2.32351**	**−1.63138**	**−0.71672**	**0.27273**
**Resilence_28**	**I took action to improve my life.**	**2.33577**	**−2.85209**	**−2.20087**	**−0.74018**	**0.33513**

**Items in** **bold** represent short form selections.

Context for all Resilience items was ‘In the past 7 days…’.

Response set was: 1 = Never/2 = Rarely/3 = Sometimes/4 = Often/5 = Always. Item Resilience_32 is reverse-scored.

All SCI-QOL Items and parameters copyright © 2015 David Tulsky and Kessler Foundation. All Rights Reserved. Scales should be accessed and used through the corresponding author or http://www.assessmentcenter.net. Do not modify items without permission from the copyright holder.

**Figure 1:**
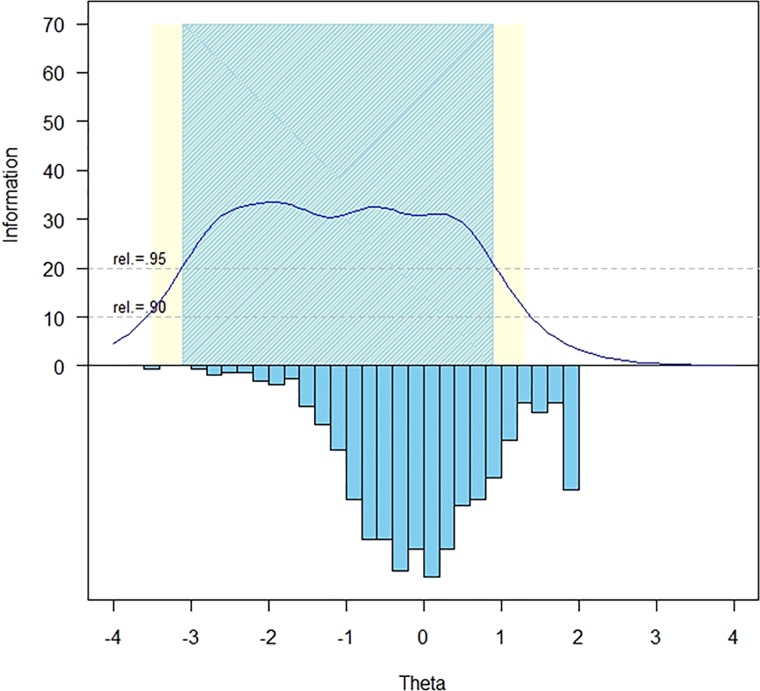
SCI-QOL resilience item bank information and precision.

The measurement precision in the theta range was between –3.1 and 0.9, which is roughly equivalent to a classical reliability of 0.95 or better (Fig. 1).

The S-X^2^ model fit statistics were examined using the IRTFIT macro program. All items had adequate or better model fit statistics (P > 0.05), with marginal reliability equal to 0.950 and no item pairs flagged for local dependence.

### Differential item functioning (DIF)

DIF was examined using *lordif*^71^ for six categories: age (≤49 vs. ≥50), sex (male *n* = 559 vs. female *n* = 158), education (some college and lower *n* = 523 vs. college degree and above *n* = 194), diagnosis (tetraplegia *n* = 388 vs. paraplegia *n* = 325), injury severity (incomplete *n* = 374 vs. complete *n* = 339), and time post injury (<1 year *n* = 196 vs. >1 year *n* = 521). Items were flagged for possible DIF when the probability associated with the χ^2^ test was <0.01 and the effect size measures (McFadden's pseudo *R*^2^) >0.02, which is a small but non-negligible effect. Overall, 10 items were flagged for at least one category based on the *χ*^2^ test, however, when the effect size measures were examined, the DIF was negligible and all 21 items were retained in the final, calibrated item bank.

### Short form selection and mode of administration

Once the SCI-QOL Resilience item bank was finalized, all items and parameters were programmed into the Assessment Center^SM^ ^72^ platform and the bank is now freely available as a computer adaptive test (CAT). Since the purpose of calibrating items using IRT is that only a subset of items needs to be administered from a given bank in order to estimate an individual's score, there is flexibility as to how the items are selected and administered. Through Assessment Center, the CAT administration parameters can be modified to reduce standard error of measurement (e.g. maximize reliability), or to reduce test burden. There is also a predetermined static short form that can be downloaded. Finally, the individual items are present and could be selected if the end user wanted to administer a specific item. These administration options are reviewed below. Item bank and short form PDFs are also available from the corresponding author.

The SCI-QOL utilizes the same default CAT discontinue criteria as the Patient Reported Outcome Measurement Information Sustem (PROMIS); namely, the CAT minimum number of items to administer = 4, maximum number of items to administer = 12, maximum standard error = 0.3. In other words, in the default settings, the CAT will always administer at least 4 items, then will discontinue when the standard error of the individual's score estimate drops below 0.3 or a maximum of 12 items is reached (and the standard error criterion cannot be met).

Alternatively, the user could change the discontinue criteria of the CAT so that it will administer additional items and obtain a more precise measurement of functioning. For instance, if the user selected an option that the CAT administers a minimum of 8 items before discontinuing, a longer test would be administered, but a more reliable score will be obtained. This would be helpful if the variable being assessed was a primary outcome variable or the assessment had higher stakes for inaccurate assessment (e.g. resource allocation based on assessments). In such cases, the user could easily modify the discontinue criteria and a more precise assessment would be administered.

However, in some cases it is neither possible (i.e. internet unavailable) nor practical (i.e. laptop/tablet computer equipment beyond budget of project) to administer items via CAT. As such, like all other SCI-QOL item banks, the project investigators utilized psychometric and clinical input to also develop a fixed, 8-item ‘short form’ version of the item bank. The goal of the short form selection process was to include the most informative items across a wide range of the underlying trait. Since all items are calibrated on the same metric, scores on the short form are directly comparable to those on the CAT or full item bank. The correlation of the short form and various CATs with the full bank are displayed in Table 3. Short forms may be administered directly within Assessment Center, or may be downloaded for administration by paper and pencil or an alternate data capture platform or system.

**Table 3:** Accuracy of variable- and fixed-length CAT and 8-Item short form: correlations with full-bank score

Mode	*N*	# Items admin			Max	%Min	%Max	Corr. w/Full Bank
		Mean	SD	Min				
Variable-Length CAT (min 4)	717	6.35	2.7	4	12	26.7	13.8	0.97
Variable-Length CAT (min 8)	717	8.64	1.4	8	12	80.6	13.8	0.98
8-Item Fixed-Length CAT	717	8	0	8	8	n/a	n/a	0.98
8-Item Short Form	717	8	0	8	8	n/a	n/a	0.97

**Figure 2:**
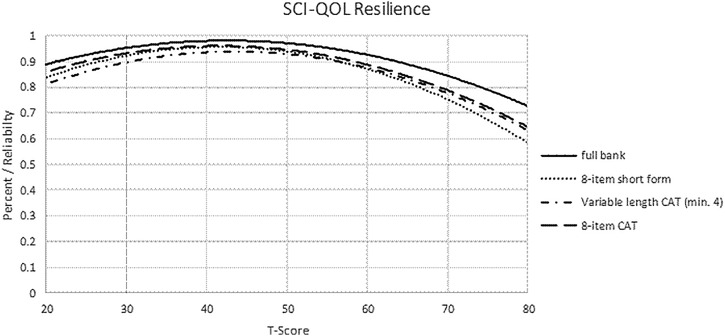
SCI-QOL Resilience: measurement reliability by T-score and assessment method. Note: CAT, Computer Adaptive Testing, which was simulated from calibration data.

To determine the degree of measurement precision and error for these assessments, we compared the reliability of the full bank, 8-item short form, and variable-length CAT with the default minimum of 4 items. Table 4 presents the mean, standard deviation, range, and standard error ranges for the various administration modes. Additionally, reliability curves for the full bank, short form, variable length CAT (min 4 items) and fixed-length CAT (8 items) are given in Fig. 2.

**Table 4:** Breadth of coverage for variable length CAT, fixed length CAT, 8-item short form, and full item bank

Mode	*N*	T Score				Standard error	
		Mean ± SD	Range	% Ceiling	% Floor	Mean ± SD	Range
Variable-length CAT (min 4)	717	50.33 ± 9.45	18.00–70.04	6.14%	0.14%	0.305 ± 0.057	0.265–0.507
Variable-length CAT (min 8)	717	50.35 ± 9.52	18.00–70.04	6.14%	0.14%	0.269 ± 0.073	0.206–0.507
8-Item Fixed-length CAT	717	50.32 ± 9.40	18.00–68.27	8.52%	0.14%	0.277 ± 0.085	0.206–0.519
8-Item Short Form	717	50.26 ± 9.25	16.80–66.40	10.75%	0.14%	0.294 ± 0.094	0.22–.58
Full Bank	717	50.37 ± 9.66	15.44–70.95	5.59%	0.14%	0.212 ± 0.083	0.152–0.491

When we compared the reliability of a CAT that was either fixed to 8 items, or a variable-length CAT with a minimum of 8 items, CAT values for both reliability (Fig. 2) and precision (Table 4) demonstrated improvement over the short form values. Based on the goal of the study and hypothesized importance of the construct of resilience (i.e. as part of the main research question or as a secondary area of interest), individual investigators will need to decide whether to administer the CAT with default settings, CAT with customized setting, or short form version of the item bank.

### Scoring

SCI-QOL Resilience item bank scores are standardized on a T-metric, with a mean of 50 and a standard deviation of 10, based on the SCI-QOL calibration data (i.e. a mean of 50 reflects the mean of an SCI population rather than the general population). All CAT administrations of the SCI-QOL Resilience item bank are automatically scored by Assessment Center. When administering the short form, whether via Assessment Center, paper and pencil, or another data capture platform, an individual must complete all 8 component items in order to receive a score. The raw score for the short form is computed by simply summing the response scores for the individual component items. The T-score and associated standard error for each raw score value are given in Table 5.

**Table 5:** T-score lookup table for Resilience SF8a

Raw score	T-score	Standard error
8	16.4	3.7
9	19.4	3.1
10	21.3	2.9
11	22.9	2.7
12	24.4	2.6
13	25.7	2.5
14	27.0	2.5
15	28.2	2.5
16	29.4	2.5
17	30.6	2.5
18	31.8	2.5
19	33.0	2.5
20	34.2	2.6
21	35.4	2.6
22	36.7	2.6
23	37.9	2.6
24	39.2	2.6
25	40.4	2.6
26	41.7	2.6
27	43.0	2.6
28	44.3	2.6
29	45.5	2.6
30	46.8	2.6
31	48.1	2.6
32	49.4	2.6
33	50.7	2.6
34	52.1	2.6
35	53.5	2.7
36	55.1	2.9
37	56.9	3.1
38	59.1	3.6
39	61.7	4.0
40	66.4	5.4

### Test-retest reliability

When comparing the SCI-QOL Resilience CAT at baseline with the CAT score from the 1-2 week follow up assessment (*n* = 245), Pearson's *r* = 0.79 (P < 0.01) and ICC(2,1) = 0.79 (95% CI: 0.74 to 0.83).

## Discussion

We developed the SCI-QOL Resilience item bank to assess the subjective experience of the process and outcome of flexibly adapting to difficult or challenging life experiences, especially highly stressful or traumatic events such as SCI. Though the construct of resilience was not on the project investigators' a priori ‘mental list’ of subdomains expected to emerge as important in the qualitative phase of the SCI-QOL project, it was clear from the individual interviews and even more so from patient focus group discussions that the concept of resilience was key to an individual's HRQOL outcomes following SCI.^63^ Resilience does not assume that major hardships are not difficult or disappear altogether, but rather that they can be tolerated, and even surmounted.

The SCI-QOL Resilience item bank fills a critical gap in existing measurement systems. While related positive psychology-focused instruments have recently been developed in PROMIS and Neuro-QOL (e.g. Psychological Adaption Scale^73^ and Positive Affect and Well-being Item Bank,^74^ respectively), none has focused specifically on the construct of resilience. Similar to PROMIS and Neuro-QOL measures, the SCI-QOL Resilience item bank was developed from direct patent input. In fact, many items were based directly off of verbatim interview or focus group statements. Moreover, the groundedness of the item bank was continually assessed by the use of cognitive debriefing interviews and large scale calibration testing with individuals with SCI. For these reasons, the SCI-QOL Resilience item bank can serve as a model that can be applied to other populations experiencing unexpected and disruptive events due to trauma, illness or injury.

### Clinical and research applications

Our primary goal was to develop a brief, flexible, and dynamic patient-reported outcomes measurement tool of resilience that is relevant to individuals with SCI and the larger SCI research community. With a new measure of resilience for the field of SCI, we can now seek to improve our understanding of the short and long term course of adaptation and adjustment following injury. Resilience is a variable that may change over time and be reflexive of positive adjustment post injury. Researchers can conduct prospective, observational and descriptive trials to better characterize specific patterns of how resilience is expressed over time, as well as identify subgroups who might experience more or less of this construct for varying reasons. By considering resilience as a ‘developable state’ that is prone to change and influence through specific psychological intervention, the SCI-QOL Resilience instrument can be used to identify critical periods along the SCI recovery trajectory for targeted clinical intervention. It also may be a mediating or moderating variable that influences how other emotional variables will change^75,76^ as people adjust to their disability. Future research should evaluate the impact of resilience as a moderator of a variety of outcomes following SCI, most notably emotional outcomes such as depression and anxiety. We also strived to assure that this new item bank would be a unique dimension in the SCI-QOL measurement system and that it would be methodologically and psychometrically connected with other SCI-QOL item banks as well as within the larger measurement systems such as PROMIS and Neuro-QOL so that a common and standardized outcomes measurement lexicon could be applied.

In addition to using this tool in research or as an outcome measure in longitudinal observational studies or therapeutic trials, it also holds promise for clinical and treatment purposes as well. For example, it may turn out to be a variable that can detect individuals who will have a more difficult psychosocial adjustment to their disability.

Due to the computerized format, this measure (along with others within the SCI-QOL system) has potential to be used as a self-monitoring screening instrument which could be self-administered using a modified touchscreen tablet computer on a serial basis (e.g. weekly/bi-weekly) during inpatient or outpatient programs to help patients learn to monitor and self-manage aspects of their rehabilitation and recovery process. Upon completing the scale, patients could be immediately presented with graphic-based feedback of their score, it's meaning to their personal trajectory and week-to-week change, as well as suggested clinical interventions that could be linked to specific score categories. Scores could also be automatically sent to rehabilitation team members (e.g. psychologist, physical therapist) who would be alerted if any significant declines were ‘triggered’ as a means of facilitating more rapid intervention. Together, these data could help inform clinical encounters between patients and providers and serve as a basis to improve communication and overall treatment satisfaction and adherence. By understanding the orientation of the patient at the start of therapy and tracking change over time, clinicians can be better armed with critical information to tailor treatment and quickly identify barriers to progress that may relate to resilience. Prior to using the Resilience items in this way, however, more work would be needed to determine clinically meaningful cut points (using standard setting methodologies) and related clinical interventions and self-management strategies that are tied to score categories. The flexibility of methods to administer the SCI-QOL Resilience item bank also provides clinicians with a cost-effective and accessible way to integrate the measure into emerging electronic medical record systems, which has widespread ramifications for use and adoption by providers, as well as the collection of important quality improvement and treatment satisfaction metrics.

## Conclusion

The final SCI-QOL resilience item bank contains 21 IRT-calibrated items. Items that were removed after testing were appraised as being conceptually related to resilience, but not an optimal way to operationally define this construct based on our working definition. Due to the flexibility of IRT-based measures, the use of computer adaptive tests is also possible with this item bank, which enables researchers and clinicians to administer only the most precise and informative items based on an individual's responses. This has implications for the use of such innovative applications in symptom monitoring and self-management post-acute care settings.

To the best of our knowledge, this is the first time that a patient-centered, modern measurement theory derived approach has been used to develop and test a resilience self-reported measurement tool specifically for individuals with SCI. Our formative development work using focus groups and interviews strengthened our understanding of resilience and its utility and importance for this population. This, coupled with the paucity of such a measurement tool in the extant rehabilitation medicine literature, makes this effort an important first step towards a greater understanding of the role of resilience and related factors in the short and long term adaptation to SCI.

## Disclaimer statements

**Contributors** All authors have contributed significantly to the design, analysis and writing of this manuscript. The contents represent original work and have not been published elsewhere. No commercial party having a direct financial interest in the results of the research supporting this article has or will confer a benefit upon the authors or upon any organization with which the authors are associated. Reprints will be available from David Tulsky (dtulsky@udel.edu).

**Funding** This work was co-funded by the National Institute of Child Health and Human Development/National Center on Medical Rehabilitation Research and the National Institute of Neurological Disorders and Stroke (NINDS) (Grant #5R01HD0054659).

**Conflicts of interest** All SCI-QOL items and parameters are © 2015 David Tulsky and Kessler Foundation. All rights reserved. All items are freely available to the public via the Assessment Center platform (http://www.assessmentcenter.net). There are currently no plans for Dr Tulsky or Kessler Foundation to benefit financially from the use of the copyrighted materials.

**Ethics approval** This work received IRB approval from the IRB at each collaborating institution.
